# Enhancement of intestinal epithelial barrier function by *Weissella confusa* F213 and *Lactobacillus rhamnosus* FBB81 probiotic candidates in an in vitro model of hydrogen peroxide-induced inflammatory bowel disease

**DOI:** 10.1186/s13104-020-05338-1

**Published:** 2020-10-20

**Authors:** Ni Nengah Dwi Fatmawati, Kazuyoshi Gotoh, I. Putu Bayu Mayura, Komang Ayu Nocianitri, Gede Ngurah Rsi Suwardana, Ni Luh Gede Yoni Komalasari, Yan Ramona, Masakiyo Sakaguchi, Osamu Matsushita, I. Nengah Sujaya

**Affiliations:** 1 Department of Microbiology, Faculty of Medicine, Udayana University, Bali, Indonesia; 2 Department of Bacteriology, Graduate School of Medicine, Dentistry and Pharmaceutical Sciences, Okayama University, Okayama, Japan; 3 School of Agricultural Technology, Faculty of Agricultural Technology, Udayana University, Bali, Indonesia; 4 Department of Cell Biology, Graduate School of Medicine, Dentistry and Pharmaceutical Sciences, Okayama University, Okayama, Japan; 5 Department of Histology, Faculty of Medicine, Udayana University, Bali, Indonesia; 6 School of Biology, Faculty of Mathematics and Natural Sciences, Udayana University, Bali, Indonesia; 7 School of Public Health, Faculty of Medicine, Udayana University, Bali, Indonesia

**Keywords:** Probiotics, *Weissella confusa* F213, *Lactobacillus rhamnosus* FBB81, Trans epithelial resistance, ZO-1 protein, Inflammatory bowel diseases

## Abstract

**Objective:**

*Weissella confusa* F213 (WCF213) and *Lactobacillus rhamnosus* FBB81 (LrFBB81) are two probiotic candidates isolated from humans in our previous study. Their functional activity on the mucosal barrier has not yet been adequately investigated. Therefore, the objective of this study was to investigate the effect of these strains on maintaining mucosal integrity in vitro. Caco-2 cell monolayers were pretreated with WCF213 and LrFBB81 before being exposed to hydrogen peroxide. The integrity of mucosal cells was evaluated by measuring the transepithelial resistance (TER), flux of FITC-labelled dextran, and ZO-1 protein distribution with the help of an immunofluorescence method.

**Results:**

WCF213 was found to significantly maintain the TER better than the control hydrogen peroxide-treated cells (*p *< 0.001), followed by the strain combination, and LrFBB81 alone (*p *< 0.05). The permeability of mucosa was also successfully maintained by the WCF213 strain. This was illustrated by the significant reduction in the flux of FITC-labelled dextran (*p *< 0.05), which was larger than that exhibited by the other groups. The ZO-1 distribution of strain-treated cells showed less disruption than hydrogen peroxide-treated cells, consistent with the TER and FITC experimental results. These findings indicate that WCF213 and LrFBB81 plays important roles in the maintenance of mucosal integrity in a strain-dependent manner.

## Introduction

The gut mucosa plays roles in protecting against luminal contents, including pathogens, and acts as a selective barrier for nutrients, water, etc. Impairment of gut barrier function occurs in gut disorders such as inflammatory bowel diseases (IBD). IBD is mostly prevalent in developed countries; however, it has recently become more common in Asia [1]. IBD is shown as a disruption of tight junctions (TJs), attenuation of epithelial resistance and increased permeability of epithelial cells due to localization or disruption of TJ protein [2]. Reactive oxygen species (including hydrogen peroxide/H_2_O_2_) are one of proinflammatory factors that can disrupt TJs and increase the permeability of gut mucosa. Factors that prevent inflammatory-mediated TJ disruption and improve gut mucosal permeability will have beneficial effects on many gastrointestinal tract diseases, including IBD. Several studies concluded that probiotics play an anti-inflammatory role by modifying the intestinal environment and subsequently reducing the severity of intestinal inflammation associated with IBD [3, 4]. Probiotics are living microorganisms that, when administered in adequate amounts, confer a health benefit on the host [5, 6]. The source of microorganisms for probiotics used in humans mainly originates from the human body, such as breast milk and faecal materials, or is cultivated from fermented dairy products that serve as human foods [7]. *Lactobacillus* spp. and *Bifidobacterium* spp. are two genera of lactic acid bacteria (LAB) used in the majority of probiotic products [8]; however, there are next-generation probiotic candidate species such as *Akkermansia muciniphila* [9] and *Faecalibacterium prausnitzii* [10]. The prominent health benefit of probiotics derives from their ability to create more favourable gut microbial niches, thereby maintaining a normal physiology of the digestive tract [6]. Any potential benefits of probiotics on the immune system, gut-brain axis, and other extraintestinal sites are considered species- or strain-specific features [6]. Recently, we isolated promising probiotic strains *Weissella confusa* F213 (WCF213) and *Lactobacillus rhamnosus* FBB81 (LrFBB81) from healthy infant faeces [11]. These two strains belong to different genera of lactic acid bacteria [12]. The latter is commonly applied as a probiotic [11], but the former has been gaining interest since this genus has a long history associated with fermented food in European sourdoughs and Korean kimchi [13–15]. Thus, the beneficial effects of *W. confusa* should be further investigated. Molecular identification of WCF213 and LrFBB81 has been performed based on 16S rDNA sequencing [11, 16]. Both of these strains have been known to have probiotic properties such as resistance to the gastrointestinal environment [11, 16], attachment to the Caco-2 cell monolayer [17], and antioxidant activity [18]. Both strains did not show haemolysis on blood agar plates [19] and did not translocate through Caco-2 monolayers [20]. WCF213 and LrFBB81 have been shown to be resistant to penicillin and vancomycin [17]; however, the vancomycin resistance was attributed to this resistance being an intrinsic factor of most lactic acid bacteria used as probiotics [21, 22]. Based on the abovementioned results, we considered WCF213 and LrFBB81 to be safe. However, their functional effect on mucosal integrity has not yet been investigated. We speculated that these two strains applied individually or in combination would protect the mucosal integrity from H_2_O_2_-induced disruption, mimicking IBD in vitro. Therefore, the aim of this study was to evaluate the protective effect of WCF213 and LrFBB81 on mucosal integrity in vitro.

## Main text

### Methods

#### Preparations of bacterial cells

WCF213 and LrFBB81, human origin-lactic acid bacteria strains that exhibit probiotic properties, were used in this study. A single strain with a cell density of 1 × 10^9^ CFU/ml or a combination of WCF213 and LrFBB81 (final cell density of 1 × 10^9^ CFU/ml for each strain) was used for the probiotic-treatment group. The bacteria were cultured in de Mann Rogosa Sharpe (MRS) agar plates (Oxoid, Basingstoke, UK) at 37 °C for 18 h under anaerobic conditions. These overnight-incubated bacterial suspensions were centrifuged at 13,000 rpm at 4 °C for 5 min. The bacterial pellets were then resuspended in Dulbecco’s modified Eagles medium (DMEM) (Fujifilm, Wako Pure Chemical Industries, Ltd., Osaka, Japan) without foetal bovine serum (FBS) according to the designated concentration.

#### Caco-2 cell lines

Caco-2 cells were passaged in DMEM with 20% FBS (Fujifilm, Wako Pure Chemical Industries, Ltd., Osaka, Japan). After passage, the cells (4 × 10^4^ cells/ml) were seeded onto 0.4-μm Transwell inserts (Corning ^®^ Inc., Corning, NY, USA) that had been pre-coated with collagen type 1 (Corning ^®^ Inc., Corning, NY, USA) and maintained at 37 °C under a 5% CO_2_ humidified air atmosphere. The medium was changed every 2–3 times per week.

#### Reagent for membrane disruption

H_2_O_2_ in DMEM without FBS was used as a TJ disruption agent.

#### Transepithelial resistance (TER) assay

The transepithelial resistance (TER) experiment used in this study (with slight modifications) has been published [20, 23]. TER was measured by using a Millicell ERS2 voltohmmeter (Merck, Millipore, Billerica, MA, USA). All cell media was changed with FBS-free DMEM before treatment. Cells were pretreated with a single strain or combination strains (treatment group) or DMEM only (control group) by adding the treatment to the apical surface of the cells. After 2 h of pretreatment, H_2_O_2_ was added to the basolateral side (final concentration 25 mM) and incubated for 4 h at 37 °C and 5% CO_2_. The TER was then measured.

#### Flux of fluorescein isothiocyanate (FITC)-labelled dextran (permeability assay)

Caco-2 cells were pretreated with WCF213, LrFBB81 or their combination for 2 h before being treated with H_2_O_2_ (treatment group) or DMEM only (control group) for 4 h. Then, 10 kDa fluorescein isothiocyanate (FITC)-labelled dextran (Nacalai Tesque, Kyoto, Japan) (final concentration 10 μM) was applied to the apical side and incubated for 3 h. Basolateral medium was collected and assayed in triplicate. The permeability of the monolayers was measured as the flux of FITC-labelled dextran from the apical chamber into the basolateral chamber and measured at 485/538 nm (excitation/emission) using a fluorometer (Ascent Fluoroscan, Thermo Scientific, Rockford, USA).

#### Caco-2 zona occludens-1 (ZO-1) immunofluorescence

The presence of zona occludens-1 (ZO-1) protein was detected using immunofluorescence as described elsewhere (with some modifications) [20]. Fourteen days post-confluence Caco-2 cells (4 × 10^4^ cells/ml) seeded onto collagen type I-coated flexiPERM^®^ (SARSTEDT AG & Co.KG, Numbrecht, Germany) were pretreated with WCF213 or LrFBB81 or their combination for 2 h and then treated with H_2_O_2_ (final concentration 25 mM) for 4 h. After incubation with H_2_O_2_, the slides were fixed with 4% paraformaldehyde in PBS for 15 min, and then washed with PBS-Tween. The cells were blocked with Blocking One Histo (Nacalai Tesque, Kyoto) and incubated for 15 min at room temperature. After washing with PBS-Tween, the specific primary antibody, ZO-1 anti-rabbit monoclonal antibody (rabbit monoclonal antibody, cat no. ab96594, Abcam) in Blocking One (Nacalai Tesque, Kyoto, Japan) and PBS-Tween were added into each well, and the plate was incubated at 4 °C, overnight. After washing with PBS-Tween, secondary antibody consisting of Alexa Fluor™ 488-goat anti-rabbit IgG (Invitrogen, Carlsbad, CA) in blocking buffer was added into the wells. The distribution of ZO-1 protein was observed as fluorescence that was visualized via fluorescence microscopy (60× oil immersion) (Biozero, Keyence, Japan). The images (60×) are representative of 10 images taken for each condition in three experiments.

#### Statistical analysis

All experiments were performed in triplicate, except where otherwise indicated. All data are presented as the mean ± SD unless otherwise specified. Statistical analysis (*independent t test*) was performed using IBM SPSS software (version 25.0, Chicago, USA). *P*-values less than 0.05 were considered statistically significant.

### Results

#### *Weissella confusa* F213 and *Lactobacillus rhamnosus* FBB81 Enhanced Mucosal Barrier Resistance in an in vitro Caco-2 Cell Model of IBD

**Fig. 1:**
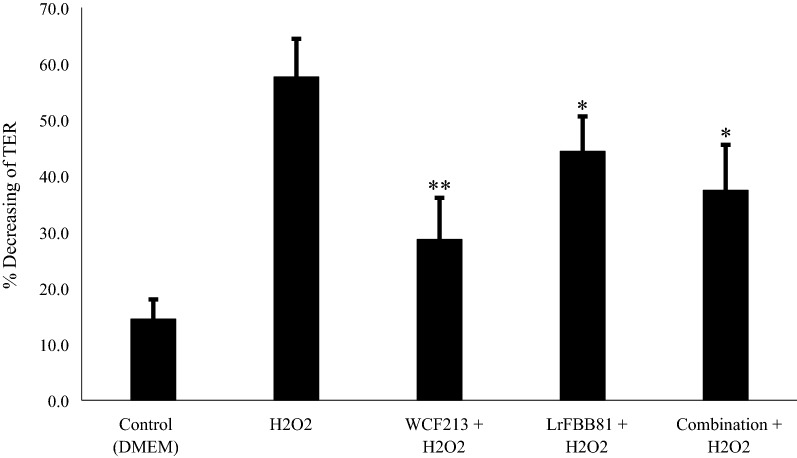
Single strain (WCF213 or LrFBB81) and combination strains pretreatment helped in maintaining mucosal integrity against H_2_O_2_ exposure. Caco-2 cells pretreated with either individual or combination strains significantly maintained TER as compared to that treated only with H_2_O_2_. WCF213 showed better effect on maintaining mucosal integrity (*p *< 0.001) than LrFBB81 or combination did. (Combination, *Weissella confusa* F213 and *Lactobacillus rhamnosus* FBB81; asterisks denote a significant difference with H_2_O_2_; Data are means of three experiments ± SD; **p < 0.001; *p < 0.05)

In this study, the effects of WCF213 and LrFBB81 on mucosal integrity in vitro were evaluated. As shown in Fig. 1, H_2_O_2_ effectively decreased the TER, indicating that H_2_O_2_ induced Caco-2 cell barrier disruption. Pretreatment with these strains, either individually or in combination, successfully diminished the H_2_O_2_-induced disruption effect on the barrier resistance of Caco-2 cell models compared with that non-strain-treated cells. Specifically, WCF213 significantly protected mucosal integrity (*p *< 0.001), better than LrFBB81 or the strain combination (*p *< 0.05) (Additional File 1: Table S1).

#### *Weissella confusa* F213 and *Lactobacillus rhamnosus* FBB81 decreased permeability in an in vitro Caco-2 cell model of IBD

**Fig. 2:**
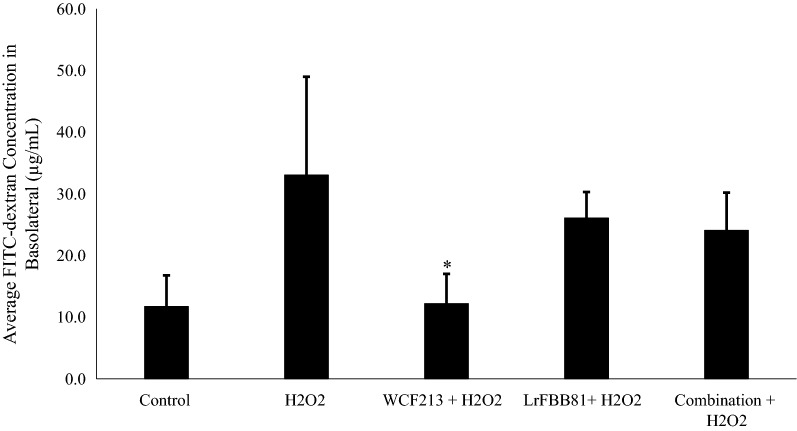
Individual strain (WCF213 or LrFBB81) and combination strains (WCF213 and LrFBB81) pretreatment aided in maintaining mucosal integrity against H_2_O_2_ exposure. Caco-2 cells pretreated with WCF213 significantly reduced of FITC dextran flux into basolateral (*p *< 0.05) as compared to that treated only with H_2_O_2_. (Combination; *Weissella confusa* F213 and *Lactobacillus rhamnosus* FBB8; asterisk denotes a significant difference with H_2_O_2_; Data are means of three experiments ± SD; *p < 0.05)

Paracellular permeability of Caco-2 cells was measured by flux of FITC dextran (MW 10,000) into the basolateral chamber. The flux of FITC-labelled dextran was lower in the strain-treated Caco-2 cell monolayer; in particular, the flux of FITC-labelled dextran in Caco-2 cells pretreated with WCF213 was significantly lower (*p *< 0.05) than that in the other groups (Fig. 2), indicating that the strains (single or combination strains) could reduce the permeability induced by H_2_O_2_, suggesting the prevention of mucosal membrane disruption (Additional File 1: Table S2).

#### *Weissella confusa* F213 and *Lactobacillus rhamnosus* FBB81 stabilized the tight junction protein in an in vitro Caco-2 cell model of IBD

**Fig. 3:**
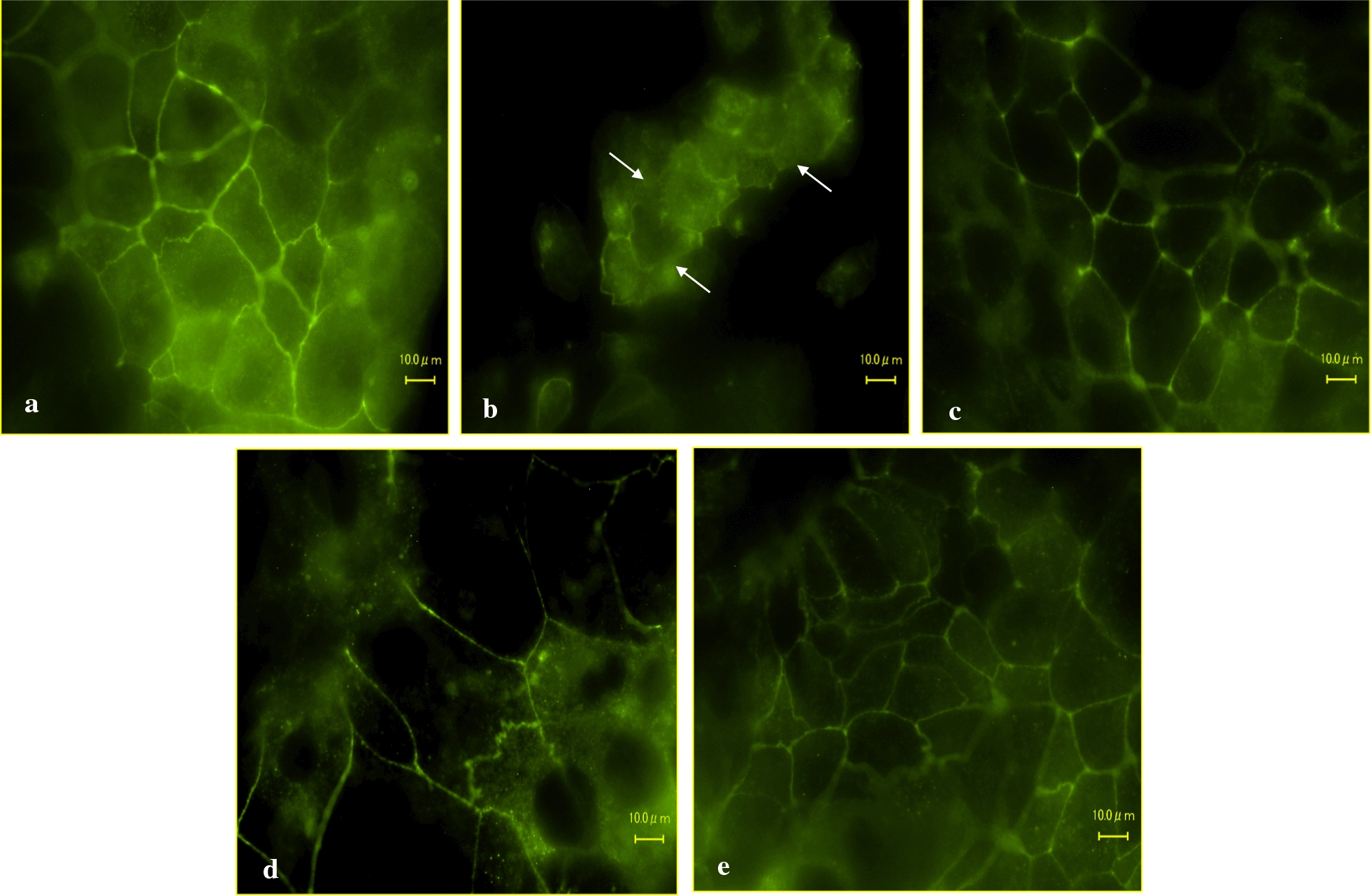
Single strain (WCF213 or LrFBB81) and combination strains (WCF213 and LrFBB81) pretreatment helped in maintaining mucosal integrity against H_2_O_2_ exposure. Caco-2 cells were untreated (control; **a**), treated with H_2_O_2_ only (**b**) and pretreated with probiotics strains (WCF213 (**c**), LrFBB81 (**d**), and combination (**e**) for 2 h, 37 °C, 5% CO_2_ before TJ disruption with 25 mM H_2_O_2_ for 4 h. ZO-1 tight junction protein was detected using immunofluorescence. ZO-1 protein of Caco-2 cells pretreated with probiotics strains (**c**, **d**, **e**) was maintained much better than that treated only with H_2_O_2_ (**b**). Cells that treated with hydrogen peroxide showed loss of their tight junction as shown as arrowheads (**b**) (observation 60× oil immersion, 10 microscope field of each treatment)

This study revealed that the strain-treated group showed more stable ZO-1 protein than the group treated with H_2_O_2_ only. In line with the TER and FITC experimental results, WCF213 was better at maintaining the stabilization of the ZO-1 protein than LrFBB81, the strain combination or H_2_O_2_ only (Fig. 3).

### Discussion

The intestinal mucosa barrier is composed of epithelial cells, the TJs between the cells, and the mucus layer [24]. Proinflammatory factors, including reactive oxygen species, damage the mucosal barrier, leading to increased paracellular permeability. Intestinal epithelial barrier dysfunction and increased permeability have been described in patients with IBD [25], which is known as dysbiosis and inflammation of the gut mucosa [26, 27]. Probiotics play a potential role not only in maintaining the composition of the microbiota but also in promoting gut mucosal integrity [28]. In the present study, we evaluated the effects of our probiotic candidate strains, WCF213 and LrFBB81, on mucosal injury caused by H_2_O_2_-induced oxidative stress in a Caco-2 cell monolayer, an in vitro model of intestinal epithelium. These strains, individually or in combination, remarkably maintained the TER, decreased the permeability, and stabilized the ZO-1 protein location at intercellular junctions. A previous study conducted by Zyrek et al. (2007) found that *E. coli* Nissle 1917, a probiotic strain, successfully enhanced transepithelial resistance in an in vitro model. The authors found that *E. coli* Nissle 1917 restored the barrier function of T84 cells after enteropathogenic *E. coli* (EPEC) infection [29]. A study using proteins produced by *L. rhamnosus* GG (LGG), p40 and p75, showed the protective effect of both proteins on mucosal integrity disruption induced by reactive oxygen species, H_2_O_2_. These proteins successfully diminished the decrease in TER after H_2_O_2_ exposure and reduced inulin flux into the basolateral membrane, which indicated that LGG treatment ameliorated the H_2_O_2_-induced disruption of TJ protein and mucosal permeability. These authors suggested that the protective mechanisms of these proteins were through protein kinase C (PKC) and mitogen-activated protein (MAP)-kinase activation [30]. A study conducted by Blackwood et al. (2017) reported that *L. rhamnosus* and *L. plantarum* significantly protected the Caco-2 cells from lipopolysaccharide (LPS)- and ethyleneglycoltetraacetic acid (EGTA)-induced disruption [23]. All these studies illustrate the potential effects of certain probiotic strains on the maintenance of mucosa integrity. Soluble peptides excreted by probiotic strains may be involved in mucosal protection against disruption agents including pathogenic microorganisms and their toxic substances. Short-chain fatty acids (SCFAs), including butyrate, produced by probiotic strains have beneficial effects on intestinal mucosa related to the proliferation and maturation of epithelium and an increase in the vascular supply, which aid in mucosal repair and play a role in TJ assembly [24, 31]. In conclusion, this study showed that WCF213 and LrFBB81 ameliorate the H_2_O_2_-induced disruption of intestinal epithelial TJs and decrease epithelial permeability; therefore, this probiotic candidate treatment represents a promising adjuvant for IBD management (Additional file 1).

## Limitation

Further investigation into the mechanism underlaying the protective effect of WCF213 and LrFBB81 on H_2_O_2_-induced mucosal injury is of interest. In this study, the strain combination showed less effectiveness on mucosal integrity than WCF213 alone. Since the effects of the probiotics were both dose and strain dependent, further studies should be conducted to optimize the dose of each strain in this combination.

## Supplementary information

**Additional file 1.** Table S1 Transepithelial Resistance (TER) of Caco-2 Cell Monolayers treated with Hydrogen Peroxide only compared with Pretreatment with Probiotic Candidates Weissella confusa F213 (WCF213) or/and Lactobacillus rhamnosus FBB81 (LrFBB81); Table S2 Flux of 10 kDa FITC-labelled Dextran (μg/mL) on Caco-2 cell monolayers treated with Hydrogen Peroxide only compared with Pretreatment with Probiotic Candidates Weissella confusa F213 (WCF213) or/and Lactobacillus rhamnosus FBB81 (LrFBB81)

## Data Availability

All data generated or analyzed during this study are included in this published article (and its additional file).

## Abbreviations

WCF213: *Weissella confusa* F213
LrFBB81: *Lactobacillus rhamnosus* FBB81
TER: Transepithelial resistant
FITC: Fluorescein isothiocyanate
ZO-1: Zona occludens-1
IBD: Inflammatory bowel diseases
TJs: Tight junctions
MRS: De Mann, Rogosa, and Sharpe
FBS: Foetal bovine serum
DMEM: Dulbecco’s modified Eagle’s medium
LPS: Lipopolysaccharide
PKC: Protein kinase C
MAP: Mitogen-activated protein kinase
SCFAs: Short-chain fatty acids
EGTA: Ethyleneglycoltetraacetic acid
