# Supplementary material for: Enhancement of intestinal epithelial barrier function by Weissella confusa F213 and Lactobacillus rhamnosus FBB81 probiotic candidates in an in vitro model of hydrogen peroxide-induced inflammatory bowel disease
Source: BMC Res Notes. 2020 Oct 20;13:489. doi: 10.1186/s13104-020-05338-1 (PMC7576752; doi:10.1186/s13104-020-05338-1)
Supplement: Supplementary file 1 — Additional file 1. Table S1 Transepithelial Resistance (TER) of Caco-2 Cell Monolayers treated with Hydrogen Peroxide only compared with Pretreatment with Probiotic Candidates Weissella confusa F213 (WCF213) or/and Lactobacillus rhamnosus FBB81 (LrFBB81); Table S2 Flux of 10 kDa FITC-labelled Dextran (μg/mL) on Caco-2 cell monolayers treated with Hydrogen Peroxide only compared with Pretreatment with Probiotic Candidates Weissella confusa F213 (WCF213) or/and Lactobacillus rhamnosus FBB81 (LrFBB81) [file 13104_2020_5338_MOESM1_ESM.docx]

**Additional file 1**

Table S1 Percentage of Decreasing of Trans epithelial Resistance (TER) of Caco-2 Cell Monolayers treated with Hydrogen Peroxide only compared with Pretreatment with Probiotics *Weissella confusa* F213 (WCF213) or/and *Lactobacillus rhamnosus* FBB81 (LrFBB81)

| Pretreatment | Mean Difference | 95% Confidence Interval of the Difference | *P-*value |
| --- | --- | --- | --- |
| WCF213 | 29.0 | 19.6 – 38.3 | 0.000** |
| LrFBB81 | 13.3 | 5.2 – 21.4 | 0.004* |
| Combination^#^ | 20.2 | 10.6 – 29.6 | 0.001* |

#Combination of WCF213 and LrFBB81

Table S2 Average of Flux of 10 kDa FITC-labeled Dextran (µg/mL) on Caco-2 cells monolayer treated with Hydrogen Peroxide only compared with Pretreatment with Probiotics *Weissella confusa* F213 (WCF213) or/and *Lactobacillus rhamnosus* FBB81 (LrFBB81)

| Pretreatment | Mean Difference | 95% Confidence Interval of the Difference | *P-*value |
| --- | --- | --- | --- |
| WCF213 | 20.9 | 3.3 – 38.4 | 0.026* |
| LrFBB81 | 7.0 | -17.7 – 31.7 | 0.450 |
| Combination^#^ | 9.0 | -12.0 – 30.1 | 0.334 |

#Combination of WCF213 and LrFBB81
